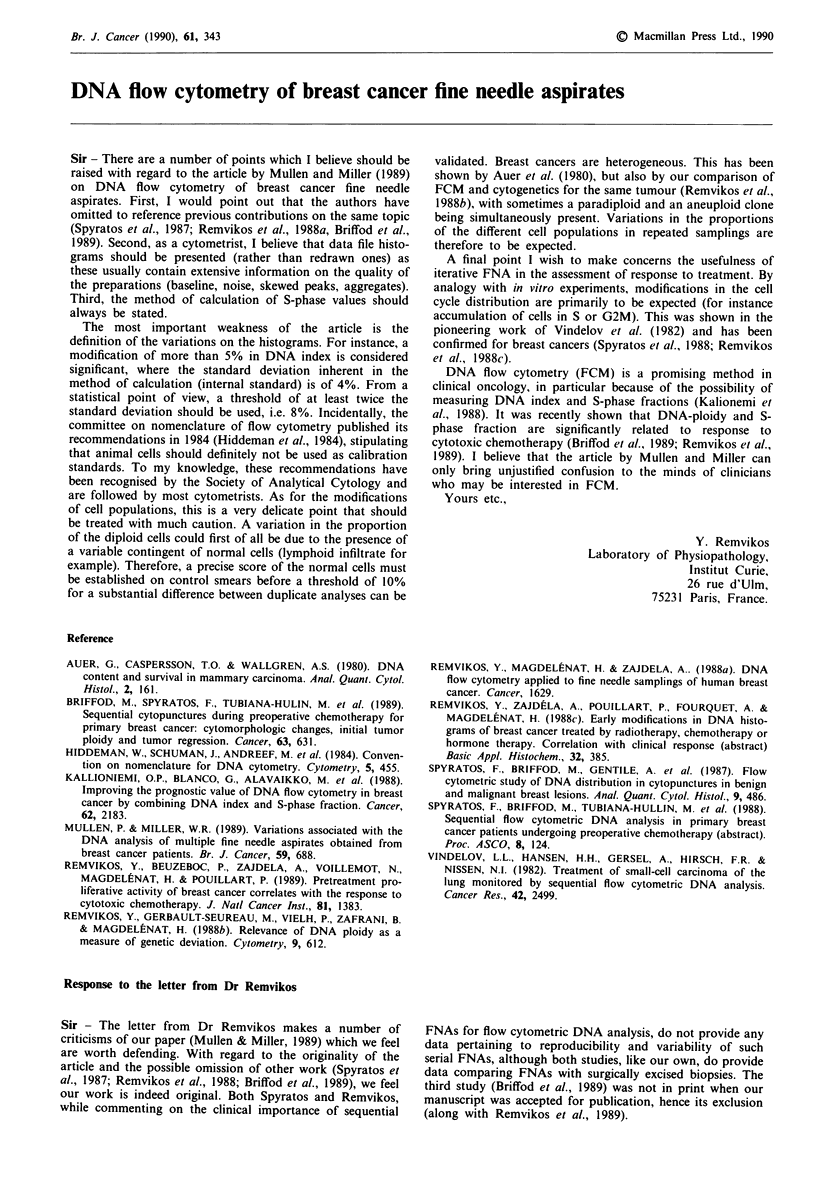# DNA flow cytometry of breast cancer fine needle aspirates.

**DOI:** 10.1038/bjc.1990.72

**Published:** 1990-02

**Authors:** Y. Remvikos


 © Macmillan Press Ltd., 1990

### DNA flow cytometry of breast cancer fine needle aspirates

**Sir** – There are a number of points which I believe should be raised with regard to the article by Mullen and Miller (1989) on DNA flow cytometry of breast cancer fine needle aspirates. First, I would point out that the authors have omitted to reference previous contributions on the same topic (Spyratos *et al.*, 1987; Remvikos *et al.*, 1988*a*, Briffod *et al.*, 1989). Second, as a cytometrist, I believe that data file histograms should be presented (rather than redrawn ones) as these usually contain extensive information on the quality of the preparations (baseline, noise, skewed peaks, aggregates). Third, the method of calculation of S-phase values should always be stated.

The most important weakness of the article is the definition of the variations on the histograms. For instance, a modification of more than 5% in DNA index is considered significant, where the standard deviation inherent in the method of calculation (internal standard) is of 4%. From a statistical point of view, a threshold of at least twice the standard deviation should be used, i.e. 8%. Incidentally, the committee on nomenclature of flow cytometry published its recommendations in 1984 (Hiddeman *et al.*, 1984), stipulating that animal cells should definitely not be used as calibration standards. To my knowledge, these recommendations have been recognised by the Society of Analytical Cytology and are followed by most cytometrists. As for the modifications of cell populations, this is a very delicate point that should be treated with much caution. A variation in the proportion of the diploid cells could first of all be due to the presence of a variable contingent of normal cells (lymphoid infiltrate for example). Therefore, a precise score of the normal cells must be established on control smears before a threshold of 10% for a substantial difference between duplicate analyses can be validated. Breast cancers are heterogeneous. This has been shown by Auer *et al.* (1980), but also by our comparison of FCM and cytogenetics for the same tumour (Remvikos *et al.*, 1988*b*), with sometimes a paradiploid and an aneuploid clone being simultaneously present. Variations in the proportions of the different cell populations in repeated samplings are therefore to be expected.

A final point I wish to make concerns the usefulness of iterative FNA in the assessment of response to treatment. By analogy with *in vitro* experiments, modifications in the cell cycle distribution are primarily to be expected (for instance accumulation of cells in S or G2M). This was shown in the pioneering work of Vindelov *et al.* (1982) and has been confirmed for breast cancers (Spyratos *et al.*, 1988; Remvikos *et al.*, 1988*c*).

DNA flow cytometry (FCM) is a promising method in clinical oncology, in particular because of the possibility of measuring DNA index and S-phase fractions (Kalionemi *et al.*, 1988). It was recently shown that DNA-ploidy and S-phase fraction are significantly related to response to cytotoxic chemotherapy (Briffod *et al.*, 1989; Remvikos *et al.*, 1989). I believe that the article by Mullen and Miller can only bring unjustified confusion to the minds of clinicians who may be interested in FCM.

Yours etc.,

Y. Remvikos
Laboratory of Physiopathology,
Institut Curie,
26 rue d'Ulm,
75231 Paris, France.

**Reference**

AUER, G., CASPERSSON, T.O. & WALLGREN, A.S. (1980). DNA content and survival in mammary carcinoma. *Anal. Quant. Cytol. Histol.*, **2,** 161.

BRIFFOD, M., SPYRATOS, F., TUBIANA-HULIN, M. *et al.* (1989). Sequential cytopunctures during preoperative chemotherapy for primary breast cancer: cytomorphologic changes, initial tumor ploidy and tumor regression. *Cancer*, **63,** 631.

HIDDEMAN, W., SCHUMAN, J., ANDREEF, M. *et al.* (1984). Convention on nomenclature for DNA cytometry. *Cytometry*, **5,** 455.

KALLIONIEMI, O.P., BLANCO, G., ALAVAIKKO, M. *et al.* (1988). Improving the prognostic value of DNA flow cytometry in breast cancer by combining DNA index and S-phase fraction. *Cancer*, **62,** 2183.

MULLEN, P. & MILLER, W.R. (1989). Variations associated with the DNA analysis of multiple fine needle aspirates obtained from breast cancer patients. *Br. J. Cancer*, **59,** 688.

REMVIKOS, Y., BEUZEBOC, P., ZAJDELA, A., VOILLEMOT, N., MAGDELÉNAT, H. & POUILLART, P. (1989). Pretreatment proliferative activity of breast cancer correlates with the response to cytotoxic chemotherapy. *J. Natl Cancer Inst.*, **81,** 1383.

REMVIKOS, Y., GERBAULT-SEUREAU, M., VIELH, P., ZAFRANI, B. & MAGDELÉNAT, H. (1988*b*). Relevance of DNA ploidy as a measure of genetic deviation. *Cytometry*, **9,** 612.

REMVIKOS, Y., MAGDELÉNAT, H. & ZAJDELA, A.. (1988*a*). DNA flow cytometry applied to fine needle samplings of human breast cancer. *Cancer*, 1629.

REMVIKOS, Y., ZAJDÉLA, A., POUILLART, P., FOURQUET, A. & MAGDELÉNAT, H. (1988*c*). Early modifications in DNA histograms of breast cancer treated by radiotherapy, chemotherapy or hormone therapy. Correlation with clinical response (abstract) *Basic Appl. Histochem.*, **32,** 385.

SPYRATOS, F., BRIFFOD, M., GENTILE, A. *et al.* (1987). Flow cytometric study of DNA distribution in cytopunctures in benign and malignant breast lesions. *Anal. Quant. Cytol. Histol.*, **9,** 486.

SPYRATOS, F., BRIFFOD, M., TUBIANA-HULLIN, M. *et al.* (1988). Sequential flow cytometric DNA analysis in primary breast cancer patients undergoing preoperative chemotherapy (abstract). *Proc. ASCO*, **8,** 124.

VINDELOV, L.L., HANSEN, H.H., GERSEL, A., HIRSCH, F.R. & NISSEN, N.I. (1982). Treatment of small-cell carcinoma of the lung monitored by sequential flow cytometric DNA analysis. *Cancer Res.*, **42,** 2499.